# Effect of Dexamethasone on Postoperative Analgesia Following the Transversus Abdominis Plane Block in Gynecological Laparotomies

**DOI:** 10.7759/cureus.73814

**Published:** 2024-11-16

**Authors:** Gözde Küçüksaraç, Kadi̇r Arslan, Ayca Sultan Sahin

**Affiliations:** 1 Anesthesiology and Reanimation, Kanuni Sultan Süleyman Training and Research Hospital, Istanbul, TUR

**Keywords:** dexamethasone, postoperative pain management, tap block, ultrasonography, visual analog scale

## Abstract

Purpose: Postoperative pain is an acute pain that begins with surgical trauma and decreases as the tissue heals. The transversus abdominis plane (TAP) block is one of the abdominal field blocks used in the treatment of acute postoperative pain after lower abdominal surgery. This study aims to investigate the effects of dexamethasone added to a local anesthetic solution on postoperative analgesia in ultrasonography-guided TAP block.

Materials and methods: Our study included female patients aged 18-80 with American Society of Anesthesiology (ASA) I-II status who had undergone gynecological laparotomy (total abdominal hysterectomy (TAH), total abdominal hysterectomy + bilateral salpingoophorectomy (TAH+BSO), myomectomy). The patients were examined in two groups, each consisting of 30 people. Patients in whom dexamethasone was added to the analgesic solution while applying the TAP block were classified as Group D and patients without dexamethasone were classified as Group B. The patients' Visual Analog Scale (VAS) scores at the 2nd, 4th, 8th, 12th, and 24th hours postoperatively; their painkiller needs in the first 24 hours postoperatively; their first mobilization time; and their total analgesic requirements in the first 24 hours postoperatively were examined.

Results: When postoperative 2, 4, 8, and 12 hours VAS scores were compared, no significant difference was found between the groups (p>0.05). Postoperative 24th-hour VAS scores were significantly lower in Group D (p<0.001). No statistically significant difference was found when additional analgesic demands were compared between the groups at 2, 4, 8, and 12 hours postoperatively (p>0.05). Additional analgesic demand at the 24th postoperative hour was significantly lower in Group D (p=0.020). When the 24-hour additional IV analgesic demands of the patients were compared, the additional analgesic demand in Group B was found to be significantly higher than in Group D (p=0.038). When postoperative mobilization hours were compared between the groups, no statistically significant difference was found between the two groups (p=0.617). When the patient's first analgesic request times were compared, no significant difference was found between the groups (p=0.617).

Conclusion: It was determined that dexamethasone added to bupivacaine in USG-guided TAP block reduced the amount of additional analgesic consumed in the first 24 hours postoperatively and reduced VAS scores. We think that adding dexamethasone to local anesthetics in the TAP block will benefit multimodal analgesia.

## Introduction

Gynecological laparotomies such as total abdominal hysterectomy (TAH), total abdominal hysterectomy + bilateral salpingoophorectomy (TAH+BSO), and myomectomy are frequently performed surgical procedures. The majority of the pain experienced after abdominal surgery stems from the abdominal wall [1]. This type of pain begins with surgical trauma and decreases as the tissue heals. Patients undergoing gynecological laparotomy typically experience moderate to severe pain after the procedure [2]. Inadequately treated postoperative pain can cause delayed recovery and mobilization, longer hospital stays, decreased respiratory function due to pain, chronic pain, increased incidence of venous thromboembolism, and patient dissatisfaction [3].

Many methods are available to treat postoperative pain. Peripheral nerve blocks are a widely used method for postoperative analgesia worldwide. A single dose of peripheral nerve block with long-acting local anesthetics generally provides potent analgesia for 8 to 14 hours [4,5]. The transversus abdominis plane (TAP) block has been used for many years to treat acute postoperative pain following lower abdominal surgeries. [6]. The effect of this block in postoperative pain management has been proven by many studies. The aim of this study is to investigate the effects of dexamethasone added to a local anesthetic solution on postoperative analgesia in ultrasound (USG)-guided TAP block.

## Materials and methods

This retrospective cross-sectional study was conducted at the Anesthesiology and Reanimation Clinic of Kanuni Sultan Süleyman Training and Research Hospital. The study was conducted in compliance with the Declarations of Helsinki for humans and approved by the Ethical Committee of Kanuni Sultan Süleyman Training and Research Hospital (İstanbul, Turkey) (approval number: 2022.01.332).

The study was prepared by examining the files of patients who underwent gynecological laparotomy (myomectomy, TAH, TAH+BSO) under general anesthesia in a one-year period between January 2022 and January 2023. Patients aged between 18 and 80, American Society of Anesthesiology (ASA) I-II, body mass index (BMI) <35kg/m^2^, who used 0.5% bupivacaine or 0.5% bupivacaine + 8 mg dexamethasone as local anesthetic were included in the evaluation process. Data such as the demographic characteristics of the patients, their need for additional painkillers in the first 24 hours postoperatively, the time when the patients first requested painkillers, Visual Analog Scale (VAS) scores at 2, 4, 8, 12, and 24 hours, time to first mobilization, nausea-vomiting and additional complications were examined and recorded on case forms. In all surgical clinics in our hospital, patients who have had nerve blocks are expected to start experiencing pain before being given painkillers. In our surgical clinics, rescue analgesics are usually administered intravenously. Rarely, oral or intramuscular analgesia may be preferred. Patients who received oral or intramuscular rescue analgesia were not included in our study.

People under 18 years of age and over 80 years of age, ASA III-IV, those with a history of local anesthesia, dexamethasone, nonsteroidal anti-inflammatory drug (NSAID), and tramadol allergy, those with local infection in the area where the block will be applied, coagulopathy, neuropathy, significant psychiatric and cognitive disorder, those with a history of substance abuse, BMI those >35 kg/m^2^, those who developed major complications during or after surgery, and those who needed postoperative intensive care were also not included.

For the TAP block, the MyLab™Six ultrasound system (Esaote S.p.A., Genoa, Italy) and 4-15 MHz linear probe are used in our hospital. The operation was performed in B-mode. TAP block was performed under sterile conditions with an 80 mm long block needle (Stimupleks B. Braun R, Melsungen, Germany). A bilateral TAP block was applied by entering between the internal oblique muscle and the transversus abdominis muscle with an "in-plane" technique under USG guidance and observing the spread of bupivacaine+saline or bupivacaine+saline+dexamethasone solution.

The patients were divided into two groups according to the local anesthetic solutions used: Group B (bupivacaine+saline group) and Group D (bupivacaine + saline+ dexamethasone group). The sample size was determined with reference to a study conducted by Akkaya et al. [7]. According to the calculation made using the G* Power 3.1 program (Heinrich-Heine-Universität Düsseldorf, Düsseldorf, Germany), a total of 36 patients were calculated, with at least 18 in each group. Sixty patients who underwent TAP block after gynecological laparotomy between the relevant dates and who met the inclusion criteria were included in the study. Statistical Package for the Social Sciences (IBM SPSS Statistics for Windows, IBM Corp., Version 21, Armonk, NY) was used to analyze the data. The suitability of the variables to normal distribution was evaluated analytically (Shapiro-Wilks test) and visually (histogram). Independent samples t-test or Mann-Whitney U test was used to evaluate quantitative data. The analysis of variance (ANOVA) test was used to analyze repeated VAS measurements between groups. Pearson chi-square and Fisher's exact test were used to analyze categorical data. The statistical significance level was accepted as p<0.05.

## Results

While the mean age was 42.16 (±6.80) years in Group B, it was 45.26 (±9.47) years in Group D. There was no statistically significant difference between the groups in terms of age, BMI, and ASA (p>0.05) (Table 1).

**Table 1:** Demographic data and some clinical features of the groups * T-test in independent groups; ** Pearson Chi-square test BMI: body mass index; ASA: American Society of Anesthesiology; TAH: total abdominal hysterectomy; TAH+BSO: total abdominal hysterectomy + bilateral salpingoophorectomy

Characteristics		Group B (n=30)	Group D (n=30)	P-value
Age	Mean ± SD	42.16 (±6.80)	45.26 (±9.47)	0.151*
	Median (Min-Max)	42.5 (26-55)	46.5 (27-67)	
BMI	Mean ± SD	25.9 (±1.76)	25.83 (±2.01)	0.892*
	Median (Min-Max)	26 (22-30)	26.0 (22-30)	
ASA	I/II	12/18	7/23	0.165**
Comorbidity	No	19 (63.3%)	11 (36.7%)	-
	Yes	11 (36.7%)	19 (63.3%)	-
Surgery Type	Myomectomy	11 (36%)	13 (43.3%)	-
	TAH	7 (23%)	3 (10%)	-
	TAH+BSO	12 (40%)	14 (46.7%)	-

It was determined that 11 (36%) patients in Group B had a myomectomy, seven (23%) patients had TAH, and 12 (40%) patients had TAH+BSO surgery. It was observed that 13 (43.3%) patients in Group D had a myomectomy, three (10%) patients had TAH and 14 (46.7%) patients had TAH+BSO surgery. There was no statistically significant difference between the groups in terms of previous operations (p = 0.452).

When 2-4-8-12 hour VAS scores were compared according to groups, no statistically significant difference was seen (p>0.05). VAS scores at the 24th postoperative hour were found to be significantly lower in Group D (p<0.001) (Table 2).

**Table 2:** Evaluation of postoperative VAS scores by groups * Repeated measures of analysis of variance (ANOVA) test; VAS: Visual Analog Scale

		Group B	Group D	P-value
2 Hours	Mean ± SD	4±1.75	3.4±1.4	0.194*
	Median (Min-Max)	4 (2-7)	3(1-7)	
4 Hours	Mean ± SD	3.4±1.03	3.03±0.88	0.156*
	Median (Min-Max)	3(2-5)	3(1-6)	
8 Hours	Mean ± SD	3.26±1.52	3.16±1.23	1.000*
	Median (Min-Max)	3(1-7)	3(1-6)	
12 Hours	Mean ± SD	2.9±1.26	2.63±1.32	0.377*
	Median (Min-Max)	3(1-6)	2(1-6)	
24 Hours	Mean ± SD	3.23±1.38	1.83±1.14	<0.001*
	Median (Min-Max)	3(1-6)	2(1-5)	

When the patients' requests for additional 24-hour IV painkillers were compared, 11 (36.7%) patients in Group B needed additional IV diclofenac once and 17 (56.7%) patients needed twice, while those in Group D needed additional IV diclofenac. Twenty (60%) patients required additional IV diclofenac once and 10 (40%) patients twice. While two (6.6%) patients in Group B required additional IV opioids, no additional IV opioids were required for the patients in Group D. The demand for additional 24-hour IV painkillers was found to be significantly higher in Group B than in Group D (p = 0.038, r = 4.286).

When the additional IV analgesic needs are compared in mg according to the groups, the mean (±SD) diclofenac requirement of the patients in Group B is 115±42.8 mg and the mean (±SD) tramadol requirement is 6.6±25.3 mg, the mean (±SD) diclofenac requirement of the patients in Group D is found to be 105±37.3 mg and the patients did not require tramadol (Table 3). No statistically significant difference was observed between the groups (p = 0.264, p = 0.154).

**Table 3:** Comparison of the need for additional IV painkillers according to groups * Mann-Whitney U Test

		Group B (n=30)	Group D (n=30)	P-value
Diclofenac (mg)	Mean ± SD	115±42.8	105±37.3	0.264*
	Median (Min-Max)	150 (0-150)	75 (75-150)	
Tramadol (mg)	Mean ± SD	6.6±25.3	0	0.154*
	Median (Min-Max)	0(0-100)	0	

No statistically significant difference was found when additional IV painkiller requests at 2-4-8-12 hours were compared between the groups (p>0.05). In Group D, the additional analgesic demand at the 24th postoperative hour was found to be significantly lower (p = 0.020) (Table 4).

**Table 4:** Comparison of the number of patients requesting IV painkillers at 2-4-8-12 and 24 hours according to groups * Pearson chi-square test

	Group B (n=30)	Group D (n=30)	P-value
2 Hours	15 (50%)	12 (40%)	0.436*
4 Hours	7 (23.3%)	7 (23.3%)	1*
8 Hours	7 (23.3%)	11 (36.7%)	0.260*
12 Hours	6 (20%)	8 (26.7%)	0.542*
24 Hours	12 (40%)	4 (13.3%)	0.020*

When the postoperative mobilization times of the patients were compared between the groups, it was seen that the patients in Group B were mobilized in an average of 6.17 hours (±0.37) postoperatively, while the patients in Group D were mobilized in an average of 6.07 hours (±0.25) postoperatively. No statistically significant difference was found between the two groups (p=0.617) (Table 5).

**Table 5:** Comparison of postoperative first mobilization hour and first analgesic request times according to groups * Mann-Whitney U Test

		Group B (n=30)	Group D (n=30)	P-value
First mobilization hour	Mean ± SD	6.17±0.37	6.07±0.25	0.617*
	Median (Min-Max)	6 (6-7)	6 (6-7)	
First request for painkillers	Mean ± SD	6.73±7.56	6±4.95	0.617*
	Median (Min-Max)	3 (2-24)	4 (2-24)	

When the hours at which patients first requested painkillers were compared according to groups, no statistically significant difference was found between the groups (p=0.617). When the groups were compared in terms of the presence of postoperative nausea and vomiting, no statistically significant difference was found between the groups (p = 196).

## Discussion

In this study, the effects of dexamethasone added to bupivacaine were examined in patients who underwent TAP block after gynecological laparotomy. The results showed that the dexamethasone-added group had a significantly lower 24-hour need for additional IV painkillers and a 24-hour VAS score than the bupivacaine group. No significant difference was detected between the groups in terms of 2nd, 4th, 8th, and 12th hours VAS scores, first mobilization time, first additional painkiller request time, and nausea and vomiting rates.

TAH, TAH+BSO, and myomectomy are frequently performed in gynecological laparotomies. These surgeries are most commonly performed for the treatment of uterine myomas. While TAH and TAH+BSO are applicable surgical methods, especially in patients with no fertility expectations, myomectomy is a feasible surgical procedure in patients with fertility expectations depending on the location or size of the myomas [8]. Hysterectomies can also be performed due to endometriosis, malignant neoplasia, endometrial hyperplasia, or dysfunctional uterine bleeding. We did not include cases operated on due to malignant neoplasms in our study.

Postoperative pain is frequently associated with incisions made in the abdominal wall. Causes of this condition include inflammation, gas, muscle spasms, and visceral pain [9]. Pain experienced in the postoperative period not only affects the patient's general well-being, but can also increase postoperative morbidity, length of hospital stay, and risk of chronic pain.

Tran et al. [10] conducted a study on 10 cadavers in which 20 cc of aniline dye was injected into the TAP under USG guidance, and the muscle structures and nerves where the dye was retained were examined. As a result of the study, it was observed that the T10-L1 nerves were affected by the 20 cc volume, and they stated that the technique could be limited to lower abdominal procedures. In our thesis study, we selected patients who received 20 cc volumes for each area.

Dexamethasone is a corticosteroid with strong anti-inflammatory and immunosuppressive properties. Systemic use of dexamethasone has been shown to reduce the incidence of postoperative nausea and vomiting and pain, and when used together with local anesthetics, it prolongs the duration of the anesthetic effect and reduces the total consumption of additional analgesics [11]. Steroids relieve pain by reducing inflammation and help reduce tissue edema. It also has a vasoconstriction effect by reducing the absorption of local anesthetics. Steroids have been used in the treatment of chronic pain for many years [12].

In a study they conducted in 2019, Gupta and colleagues [13] applied postoperative TAP block after cesarean section to a group of patients. While dexamethasone was added as an adjuvant in one group, only ropivacaine was used in the other group. In the dexamethasone group, 8-12-24th hour VAS scores and the need for additional painkillers in the first 24 hours were significantly lower than in the ropivacaine group. In our study, we did not find a significant difference between the groups at the 8th and 12th hours, but the 24th-hour VAS scores and the need for additional 24-hour painkillers were significantly lower in the dexamethasone group. The difference between VAS scores may be due to ropivacaine.

In a study conducted by Kartalov et al. [14] in 2015, patients who underwent inguinal hernia surgery were divided into three groups. TAP block was not performed in the first group, TAP block was performed with only ropivacaine in the second group, and TAP block was performed by adding dexamethasone to ropivacaine in the third group. When the VAS scores of the 2nd, 4th, 6th, 12th, and 24th hours were examined, it was seen that the VAS scores of the third group were significantly lower than the first and second groups at each hour.

In the study conducted by Ammar et al. [15] in 2012, they divided the patients into two groups after abdominal hysterectomy. While one group underwent a TAP block with only bupivacaine, the other group underwent a TAP block with the addition of dexamethasone to bupivacaine. When the VAS scores of the two groups were compared at 1, 2, 4, 12, 24, and 48 hours, the VAS scores of the dexamethasone group at 1, 2, 4, and 12 hours were found to be significantly lower than the bupivacaine group, while no significant difference was observed between the VAS scores at 24 and 48 hours. The total morphine requirement of the dexamethasone group in the first 48 hours was found to be significantly lower than the bupivacaine group.

The results of studies on the effect of adding dexamethasone to the TAP block on the duration of analgesia are quite diverse. Some studies claim that analgesia is prolonged for 20-24 hours [13,14] while others suggest that it is effective for up to 12-16 hours [15,16]. In our study, we observed that significant analgesia continued for up to 24 hours. This result is consistent with other studies in the literature regarding the decrease in the use of additional painkillers.

The study by Knezevic et al. [17] in 2015 suggests that dexamethasone should be used in doses higher than 0.1 mg/kg to contribute to postoperative pain. In our study, we selected patients who were administered a total of 8mg of dexamethasone, 4mg on each side.

Although very rare, TAP block has been associated with a number of complications. These complications include hematoma, vascular injuries, intra-abdominal organ perforation, infection, and local anesthetic systemic toxicity. No complications related to the TAP block were observed in our study.

Limitations of the study

Among the limitations, it can be said that there is no control group and the patients are not monitored after the first 24 hours. TAP block is generally performed through a lateral approach in our clinic. However, it may rarely be performed through an anterior or posterior approach. VAS score is a subjective assessment. It may vary depending on the pain thresholds of the patients and the person evaluating.

## Conclusions

When the 2-4-8-12th hour VAS scores and additional rescue analgesic requirement were compared between the groups, no statistically significant difference was found. However, the 24th-hour VAS scores and additional rescue analgesic requirement were significantly lower in the dexamethasone group. It was observed that the addition of dexamethasone to bupivacaine as a local anesthetic in TAP block reduced the use of opioids and additional analgesics in the first 24 hours postoperatively. No significant difference was found between the groups in terms of nausea, vomiting, and first mobilization time. It was concluded that dexamethasone can be added to postoperative multimodal analgesia and applied in daily practice. Although the use of dexamethasone as an adjuvant in TAP blocks seems appropriate, studies with larger groups are needed.
